# Possibility Routes for Textile Recycling Technology

**DOI:** 10.3390/polym13213834

**Published:** 2021-11-06

**Authors:** Damayanti Damayanti, Latasya Adelia Wulandari, Adhanto Bagaskoro, Aditya Rianjanu, Ho-Shing Wu

**Affiliations:** 1 Department of Chemical Engineering and Materials Science, Yuan Ze University, 135 Yuan-Tung Road, Chung-Li, Taoyuan 32003, Taiwan; damayanti@tk.itera.ac.id; 2 Department of Chemical Engineering, Institut Teknologi Sumatera, Jl. Terusan Ryacudu, Way Huwi, Kec. Jati Agung, Lampung Selatan 35365, Indonesia; latasyadeliaw@gmail.com (L.A.W.); adhanto.118280076@student.itera.ac.id (A.B.); 3 Department of Materials Engineering, Institut Teknologi Sumatera, Jl. Terusan Ryacudu, Way Huwi, Kec. Jati Agung, Lampung Selatan 35365, Indonesia; aditya.rianjanu@mt.itera.ac.id

**Keywords:** textile recycling, mechanical recycling, pyrolysis, enzymatic hydrolysis, ammonolysis, glycolysis, IoT, sorting identification

## Abstract

The fashion industry contributes to a significant environmental issue due to the increasing production and needs of the industry. The proactive efforts toward developing a more sustainable process via textile recycling has become the preferable solution. This urgent and important need to develop cheap and efficient recycling methods for textile waste has led to the research community’s development of various recycling methods. The textile waste recycling process can be categorized into chemical and mechanical recycling methods. This paper provides an overview of the state of the art regarding different types of textile recycling technologies along with their current challenges and limitations. The critical parameters determining recycling performance are summarized and discussed and focus on the current challenges in mechanical and chemical recycling (pyrolysis, enzymatic hydrolysis, hydrothermal, ammonolysis, and glycolysis). Textile waste has been demonstrated to be re-spun into yarn (re-woven or knitted) by spinning carded yarn and mixed shoddy through mechanical recycling. On the other hand, it is difficult to recycle some textiles by means of enzymatic hydrolysis; high product yield has been shown under mild temperatures. Furthermore, the emergence of existing technology such as the internet of things (IoT) being implemented to enable efficient textile waste sorting and identification is also discussed. Moreover, we provide an outlook as to upcoming technological developments that will contribute to facilitating the circular economy, allowing for a more sustainable textile recycling process.

## 1. Introduction

The improvement of living standards around the world due to economic development could be affected by the textile industry. The textile industry faces huge challenges related to environmental issues. In 2018, the total global textile production was 105 million metric tons [1,2,3,4]. In total, up to 64% of the textile fibers that are produced are produced from the petrochemical industry. The rest of the fibers, 36%, are shared by cotton, 24%, cellulosic fibers, 6%, and wool 1% and natural fibers. With its present fast fashion business model, which is defined by mass production, variety, agility, and affordability, the apparel industry has made a significant contribution to the amount and rate of trash generation [5]. The volume of textile waste that is primarily being disposed of in landfills or incinerators is continuing to significantly increase [6]. Less than 1% of the material used to produce clothing was recycled into new clothing [7].

Textile waste can be categorized into pre-consumer and post-consumer waste. Furthermore, there are several materials that can be used to produce fabric: (i) natural materials; the natural resources used to produce it, e.g., wool and silk (protein fibers); cotton, linen, and hemp (cellulosic); (ii) regenerated material: produced from natural polymers through processing, such as rayon, viscose, cuprammonium, and acetate (semi-synthesis fiber); and (iii) synthetic material: materials that come from the petrochemical industry, e.g., polyester and nylon [8]. Natural materials are environmentally friendly. Synthetic fibers are petrochemical-based products that are produced from materials that are not sustainable and that require enormous amounts of energy to be produced [9]. Textile waste can be recycled and utilized in secondhand stores to create value-added items. The circularity approach is currently being used in the textile reuse and recycling process, which is characterized by changing from the old linear paradigm of “take, make, and trash” to a circular model, where fiber, fabric, or garments can be used to their maximum potential [6,10]. Currently, recycling technologies have five problems: (i) lack of commercially viable recycling technologies for a low-grade textile fraction; (ii) lack of mainstreamed, up-scaled processes and know-how to separate fiber types from the mixed blends and composite structures; (iii) costly recovery process; (iv) the fact that the recycling end-market is dominated by low-quality materials and blends; (v) and costly logistics and the low availability of textile recycling plants on both local and regional levels [11]. In addition, textile recycling techniques should be pass the Life Cycle Assessment (LCA). The LCA suggests a critical area in the textile supply chain, specifically, it emphasizes recycling techniques and transportation distances between the textile recycling plant and waste collection areas due to the amount of energy that is consumed during transport [12,13,14,15].

Due to the enormous waste and pollution caused by the phenomena of fast fashion, the textile sector faces tremendous environmental and resource challenges [16]. The transition to a circular economy (CE) is already underway, and it offers an effective option for resource scarcity and waste disposal issues [17]. Sorting for textile material identification and separation takes place before textile waste is recycled due to the complex material in textile fibers [18]. Moreover, in the future, the large-scale separation and sorting of textile waste, regardless of the strategy that is selected, quick and reliable methods to accurately quantify the polyester content in cotton, polyester, and other textile material compound mixes are urgently needed [19]. Specific materials should be recycled, e.g., medical textiles, which are all fiber-based products and structures that are utilized for emergency treatment and clinical, surgical, and hygienic purposes [20]. The process of recycling textile waste can be easily achieved by Internet of Things (IoT) technology. IoT can assist in collecting the data provided by various sensors, including smart meters, in connecting stakeholders across the value chain [21]. Furthermore, IoT needs data collection due to the fact that smart sensor and RFID technologies require big data [22]. Fatimah has been introduced to modern technologies such as Indonesia’s IoT-based sustainable CE approach; waste management has also benefited from IoT technology [23].

Identifying textile materials is the most challenging task in the textile recycling process because of the complicated structures of fabrics and textiles. Cellulose polyester mixtures are among the most popular multi-component garments on the marketplace and can be used to customize material attributes such as moisture absorption, wrinkle resistance, and wearing comfort [24]. There are some promising methods that can be used to analyze the complex components of different textiles. For example, applying near-infrared (NIR) spectroscopy and chemometrics for textile analysis was reported over two decades ago [25]. The cotton component, which comprises mixed cotton and polyester, was determined by genetic algorithms [25]. In addition, nuclear magnetic resonance (NMR) has been used to predict the proportion of cellulose and polyester in textile blends [24]. Nevertheless, solid-state NMR can show lower sample throughput and efficiency. Still, it allows for more precise measurements that are not influenced by factors such as particle size, dyes, or surface structure [26]. On the other hand, the nature of the sample surface, such as particle size, brightness, color, moisture content, and coating chemicals, has a significant impact on the NIR signals that are produced [27].

Polyester and cellulose performed very differently in terms of behavior, especially when considering differences such as thermal stability and hydrophilicity. The diversity of properties in these textiles allows them to be reprocessed and separated in different ways, such as through depolymerization, re-polymerization, and spinning [24]. Therefore, solving these problems requires a comprehensive analytical toolkit for the qualification and identification of textile materials [28]. Textile can be recycled through mechanical, thermal, and chemical methods. Textile recycling mainly entails the reprocessing of post- or pre-consumer waste fabric in order for it to be reused in new textile or non-textile products. The three types of textile recycling routes are the dissolution of natural fibers, the chemical degradation of polymeric fibers, mechanical treatment (pretreatment), and thermal treatment [29].

A research gap exists in the field of textile recycling technology, and it is possible for textile recycling technologies to be implemented based on the CE principle. Current technologies such as the IoT and big data can be used to enable the efficient collection of textile waste, identification, and recycling. Still, there are some challenges to recycling textile waste due to different materials that are added to fabric and textiles to make fashion items. Therefore, the strategies that can be used to recycle textile fibers, such as mechanical recycling, chemical recycling (pyrolysis, enzymatic hydrolysis, hydrothermal, ammonolysis, gasification, glycolysis), and decolorization technology in textile recyclizing, are reviewed to minimize the textile waste that ends up being incinerated or in landfills.

## 2. Circular Economy of Textile Recycling

A CE is a concept where material production can be redesigned. It utilizes resources to produce, use, and dispose of in favor of as much reuse and recycling as possible [8]. The CE approach has an environmental, industrial economic, and ecological basis [30,31,32]. There are some barriers impeding the implementation of CE in the fashion industry due to the various fashion styles, aesthetics, and the roles of shoppers. The regulations and policies regarding the environment and sustainability of CE need to be considered [19]. It has also been determined that the textile business also has a detrimental impact on society because it consumes enormous resources such as oil, carbon, and water [33,34]. Apart from that, it eliminates the over-generation of waste and obtains the total value of products by focusing on the CE through giving products a longer life and through reusing materials [8]. The CE is driven by three main methods and approaches (reduce, reuse, and recycle); all traditional waste managements are practiced [35].

**Figure 1:**
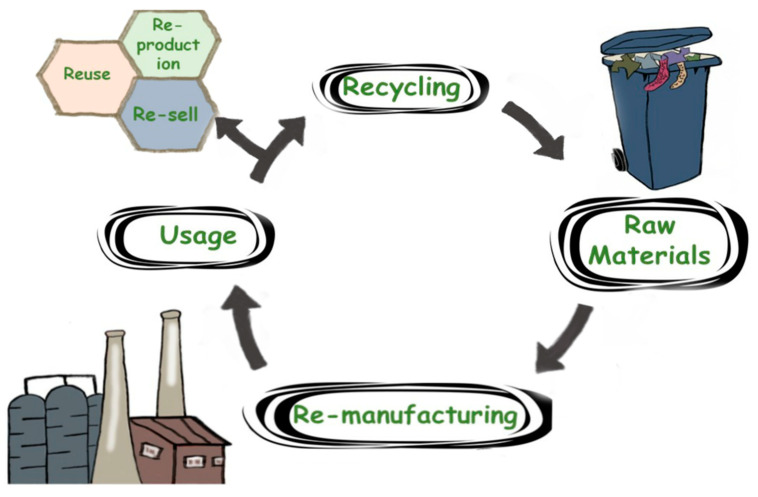
The critical point of CE.

Fabric waste reduction includes all of the manufacturing stages (including a minimum of feedstock) and various stages of use and consumption. The concept of the reuse stage is that textile materials are easily recyclable or repurposed for different types of applications. In addition, increasing reuse and repurposing will reduce the amount of textile materials that are needed for production [18,36]. Figure 1 shows this critical point in the textile CE. It is vital to have a CE because it emphasizes seeking scientific solutions to complete the loop. Waste is reduced at the source and is recycled back into the economy for reuse rather than production and consumption being stopped due to garbage disposal [37]. The CE cycles includes recycling, reuse, and remanufacturing. Textile reuse involves various methods for extending the useful life of textile items by redistributing them to a new buyer [38]. Nevertheless, the secondhand clothing markets will end up exporting these secondhand textiles to emerging market countries. On the other hand, CE emphasizes local consumer reuse, and the retailers must contribute to this circular system. This system can be applied to introduce new customer segments and new potential for reuse models [39].

Remanufacturing is used in the fashion and textile industry to describe the process of reconstructing worn clothes to create new garments by considering the quality of the textile product or the customer value [40,41]. In the early 21st century, the consideration of textile remanufacturing gained popularity because fashion designers and entrepreneurs were more aware of the sustainability of the post-consumer raw materials and textile waste [42,43]. The reasonable prices and the remanufacturing of the textile industry in some developing economies, such as in China, have been more competitive [44]. Furthermore, by using an eco-efficient value creation (EVC) model integrated with analysis cost, eco-cost, and retail value, remanufactured goods have a lower eco-cost due to their reduced materials and pollution. An EVC has a positive effect on the cost-benefit [45].

Due to the textile sector, recycling is the principal environmental polluter, and textiles have complicated the manufacturing process. Furthermore, the microfibers in textile waste harm the oceans, and the textile industry contributes to this pollution. Globally, textile waste increases, and recycling could help reduce new material waste [14]. Mechanical and chemical recycling are two primary types of recycling technologies. Mechanical recycling via melt-extrusion is a technique that is used to extract fibers from waste materials, allowing them to then be spun into yarns. Therefore, to obtain great yarn strength and fineness, this technique must be applied to virgin fibers.

On the contrary, chemical recycling occurs when the polymers of textile waste are depolymerized into small monomers and/or are re-polymerized to make new fibers. The chemical recycling of cellulose fabric such as cotton or viscose also uses the dissolution approach. At the same time, organic solvents apply ionic liquids to dissolve cellulose polymers [45,46]. In addition, the valorization process offers another potential textile recycling method. Li et al. developed the term “textile waste valorization”, which is related to the combination of “textile waste recycling and hydrolysis process”. This process degrades the complex polymers found in textiles in order for them to become various value-added products that are supported by microorganisms such as enzymes. This kind of method is inspired the CE approach, which aims provide multiple solutions for recycling textile materials [47].

Textile material recycling affects a variety of substances and contributes significantly to current social obligations. The recycling process allows businesses to make a more significant profit by avoiding textile waste being dumped in landfills. Meanwhile, the textile recycling contributes a positive impact on social and environmental issues, e.g., gives job opportunities to workers who are unemployed or underemployed, philanthropic commitments, and the expansion of reusable textile to the countries around the world that need proper cloth [48,49].

There are many challenges when transitioning the textile industry to a circular economy: (i) the market for recycled textile products is still limited; (ii) there is a lack profitable circular business models on the market; (iii) there are challenges in having supply chain partners work together to create innovations; and (iv) the quality of recycling of textiles of a relatively low grade is pricey in the short-term, with low economic benefits [50]. There are some fundamental strategies for recycling business models (RMBs); for instance, recycling process, partnership, and scale up the business models (BMs). Furthermore, approach RMBs are becoming more practicable. Collaboration between various stakeholders and across a variety of industry sectors is needed [51]. In addition, the participation of waste companies and educational institutions in investigating several options for closing material loops and identifying waste streams is a crucial change agent to facilitating the textile CE transformation [52].

## 3. Starting with Municipal Waste to Textile Trash in Applied IoT

Sustainability is also required in the waste management sector. It starts with the manufacturing process to the disposal phase. A loop feedback system for sustainable waste management comprises several parts, such as process activity and waste diversity [23,53]. The direct regulations of the European Union’s CE action plan will prohibit the disposal of unsold textile products and a restriction on single-use products, and according to the Recycling and Waste Reduction Act 2020 with replaced the product stewardship Act 2011 in Australia, there are textile regulations that are related to the ambition of separate textile collection by 2025. A new French legislation has established a command-and-control prohibition on the destruction of various unsold products, including fashion items. This legislation requires that new products use a minimum amount of recycled material [54].

Furthermore, to support the current regulation and policy of waste textiles, digital innovations have been adopted. Recently, digital innovation and industry 4.0 have positively influenced CE transformation, such as data-driven product lifecycle analysis [55]. Industrial revolution 4.0 is achieving waste treatment sustainability, with waste treatment becoming more practicable, dependable, clear, efficient, and optimal due to the application of IoT, digitalization, and information and communication technology (ICT). ICT and IoT implementation can minimize the time and resources needed to improve waste management effectiveness and to move toward more sustainable and intelligent systems [56]. In addition, these methods will be selected as the best treatment technologies for the waste material in terms of technical, economic, and environmental feasibility. IoT and ICT can be combined to gather, analyze, and share data [23].

Digitalization can be improved in product/raw materials tracing and tracking by providing real-time data on product availability, location, and condition. At the same time, sensors and digital platforms can enhance the product life and can create CE solutions more effective and efficient [55]. IoT-enabled manufacturing is related to advanced concepts. Traditional manufacturing resources can be transformed into smart manufacturing objects (SMOs) that have the ability to interact, sense, and interconnect with one another to perform manufacturing logic [57]. The fundamental issue with these technologies is the sharing and collecting of real-time data, including wireless communication standards and radio frequency identification (RFID). Physical production flows, including material transportation, and associated information flows, such as the visibility and traceability of different manufacturing operations, can be easily connected utilizing RFID technology [58,59].

Cloud computing technology is a data management solution, and it can be used as a replacement for local servers [60]. It has resulted in a massive scale of data sharing among several users and has become the latest data management standard [61]. These processes and resources must be handled intelligently in cloud manufacturing technology because the product life cycle includes the start of the manufacturing process, and includes design, manufacture, and maintenance [62]. Resources can be controlled and managed automatically by applying various IoT technologies, making digital sharing easier. Underpinning technologies are typically service-oriented, and cloud manufacturing complements the conceptual framework of cloud manufacturing [63].

**Figure 2:**
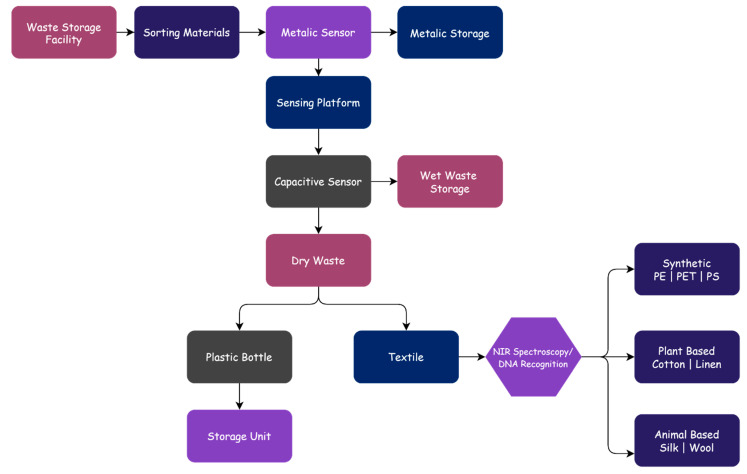
The sorting and identification of textiles using IoT.

The design approach for the sorting and identification of textile waste is based on the IoT in Figure 2. Waste materials are sorted through a funnel and a single metallic sensor. The metallic sensor detects any metal compounds that are going through the funnel. The metal materials go into metal storage. If the object does not contain any non-metal materials, then it is placed on the sensing platform. The capacitive sensors sense the object while it is on the sensing platform based on its moisture content. Then, the materials that have moved through capacitive sensors are divided into two sections: plastic bottles and textiles. Furthermore, the sorted textile waste will undergo several sorting and identification processes due to the complex mixture of materials [64].

On the other hand, manual sorting can be applied. Mechanical machines will transport the materials to a manual sorting platform by conveyors for preliminary sorting, which will sort the hazardous materials, bottles, large glasses, cotton, and textiles to be recycled. Each material will be separated based on its characteristics and will then be delivered to each storage bin. Each storage bin is equipped with a weight level sensor that sends the general information into the database. The database will receive and store all of the data related to the system’s waste workflow. According to the performance of the trash that is collected and the existing processing technology, the database will suggest treatment options [23].

Yu et al. developed a hybridized intelligent framework (AIHIF) based on artificial intelligence (AI) for automated recycling to improve trash management. By combining machine learning and graph theory, the system enhances waste-collecting over a short distance. Furthermore, AI design technology can be supported through various approaches that can be adapted to specific interest groups, collecting their data and increasing ecological planning and urban management production, precision, and efficiency [65]. Enevo has already developed recycling systems and innovative waste treatment technologies with the Enevo One software. The software contains ultrasonic wireless sensors. Waste collection optimization has been performed not only by cloud service in the background but also by the web interface used for Enevo One and the sensors themselves. Enevo’s sensors are compatible with more than 100 container types. The following waste types are currently supported: mixed, textile, glass, metal, and waste electrical and electronic equipment (WEEE) [66]. In addition, Stack4things was created by the mobile and distributed systems lab at the University of Messina in Italy. The OpenStack add-on controls sensing and actuation resources, including remote control, virtualization, and network overlays. The main objective is applied through cloud computing to manage data and changes to a software as a service (SAaaS) perspective, in which developers or end-users are provided with actual or virtualized sensing and actuation resources [67].

## 4. Sorting and Identification of Textile Waste

**Figure 3:**
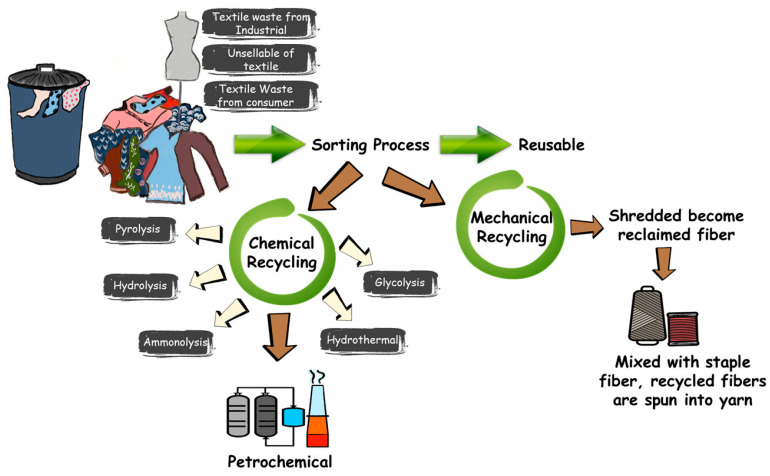
Mechanisms of possible textile waste recycling routes.

The dilemma for textile recycling is sorting and identifying textile materials due to the complex structure of textile polymers. In addition, the majority of textile materials comes from fiber, and the main challenges are sorting and recycling due to the complicated fabric of the textile. The fabric waste is commonly mixed with other materials, such as buttons, zippers, or other decorations [68]. There are various ways to identify textile materials, including iso-standardized quantification methods based on distinct dissolution behavior (ISO 1833-1, for example) and morphological variations found using microscopy [69]. Figure 3 shows the source and mechanism of textile recycling, a common source of textile waste from consumers, and unsellable textile waste from the textile industry. In addition, the sorting and collecting of textile waste is not able to be achieved manually, which would improve material quality assessment [70].

The thermal properties of textile materials are essential qualities of these materials and are regarded as the most crucial consideration when describing the comfort properties of apparel [71]. In addition, several different thermal properties have been demonstrated in numerous research studies, such as in textile fiber polymers, which are semi-crystalline, whose thermal conductivity is affected by the molecular structure, crystallization level, and molecular chain mobility of amorphous regions, etc. [72]. One of the methods that can be used to analyze the thermal properties of textile waste is by thermogravimetric analysis (TGA). The data resulting from TGA are related to the thermal stability and degradation degree from the fibers. TGA is a simple method that can be used to obtain data on the thermal behavior of a textile before that textile waste is pyrolyzed in the industry [73]. Therefore, some real-time technology is required to develop the textile material recognition rate.

Table 1 indicates the recognition rate using different types of analyzers. A single type of textile waste polymer is easier to convert into small molecules with a higher value than when other types of waste polymers are presents. Recently, near-infrared (NIR) technology has been applied to sort textile waste. NIR spectroscopy can be implemented automatically by convolutional neural network machine learning (CNN) followed by wavelength recognition that has been transformed through image classification. Then, the surface area of the spectrum is converted and modified by Textile Recycling Net (Tr-Net), and the images are sorted using the softmax classifier, allowing the qualitative of textile compositions to be obtained [74,75].

Wilts et al. developed a new AI technology to sort textile waste from municipal waste. This robot with AI could be applied with a recognition ability of more than 95%. On the other hand, textile waste recovery was not achieved, with only 13% of textile waste being recovered due to the complicated structure of materials [76]. Xiong designed a cheap near-infrared device to sort the seven types of plastics using machine learning. Machine learning could identify the plastic types with 90–99% certainty [77]. Berghmans et al. studied infrared spectrometer techniques to identify the post-consumer and industrial carpet waste. The carpet waste materials came from polyamide-6, polyamide-66, polyester, polypropylene, and wool. The complex of the carpet materials can be determined by analyzing the radiation passing through the fibers. The range of wavelengths near and mid-infrared was (800–2500 mm) and (2500–25,000 mm), respectively [78].

Riba et al. used an ATR-FTIR spectrometer (Attenuated Total Reflection–Fourier Transform Infrared) to sort and classify textiles based on the type of polymer materials in that textile. Mathematical modeling was applied to more accurately predict the data from the IR spectrum. The recognition rate percentage achieved by the mathematic algorithms (principal component analysis, canonical variate analysis, k-nearest neighbors algorithm (k-NN algorithm)) could detect synthetic and natural fibers, such as polyamide, viscose, polyester silk, linen, cotton, and wool, with an accuracy of up to 100% [86]. Furthermore, Peets et al. evaluated the capability of reflectance-FTIR (r-FTIR) to recognize textile fibers using an FTIR micro-spectrometer. In reflectance mode, the FTIR micro-spectrometer several reflectance spectra from textile fibers can be collected, and these spectra contain enough characteristics to create classification models and to identify unknown textile samples. Nevertheless, r-FTIR is a good approach for analyzing many textile samples quickly, easily, non-destructively, and non-invasively [87]. Morgado et al. identified PE, PET, PP, and PS using micro-ATR-FTIR spectra with known uncertainty [88].

The following three primary characteristics are reflected in the recognition and classification accuracy: (1) when physical properties, such as fabric color, yarn thickness, diameter, orientation, and uneven light, are considered, the model remains robust; (2) a model with fewer parameters should use the transfer learning technique, making it more computationally efficient; (3) the suggested model does not use handcrafted features, instead relying on a fully automated end-to-end architecture to extract and classify features [82].

## 5. Mechanical Recycling of Textile

Mechanical textile recycling has been developed since the industrial revolution began. Furthermore, mechanical recycling is one of the easiest recycling methods can be used with a low-cost budget. Textile waste is classified by the type of material and the color achieved through mechanical deconstruction; for instance, wool converts into useable yarn (re-woven or knitted) when carded yarn is spun with mixed shoddy [89,90]. Mechanical recycling can be categorized into several different methods based on the degree of breakdown that is undergone by the recovered material, such as fiber, fabric, polymer, and monomer recycling. The mechanical recycling applications in the wiper, fiber material, and prespun fiber industries could produce new fabrics for products [91].

On the other hand, there are few mechanical recycling options for post-consumer textile waste fabrics due to the variety and amount of fiber elements in one textile waste garment; mixed textiles cannot be mechanically recycled effectively. The shredding technique reduces the length of post-consumer textile waste garment fibers. The resulting short fibers are of a lower quality and strength than virgin cotton fibers, and they are frequently combined with virgin fibers to improve quality [11,13,92,93].

The metallic elements are removed when post-consumer textiles are converted to wipers. Textiles from the post- and pre-consumer markets can also be used as a raw material for filler products, including flocking, insulation, and nonwovens as well as shoddy fibers for recycled yarns [94]. A defector is developed with the mechanical ripping and carding processes during the production process. The first step of the defector process requires the feedstock to be shredded by the rotation crusher and sorted based on the type of fiber. Secondly, the fiber is crushed through correct fraction sizes and processes with the card oil. Furthermore, the defibering process is a three-phase mechanical ripping carding process. The materials are needled and folded to create a fiber blanket. In the needling process, other materials could be added to the product, e.g., plastic material. Then, the fiber blanket is trimmed to the correct size and is packed [95]. On the other hand, some textile materials cannot be recycled by mechanical process due to the low quality and difficulty of reprocessing these materials through the melting process to achieve high-value products, for instance, cotton or a mixture of synthesis fibers [90]. In addition, mechanical shredding harms natural fibers more than synthetic fibers in blended fabrics such as polyester–cotton. Chemical recycling becomes an alternative to recycle both fiber types [96].

**Figure 4:**
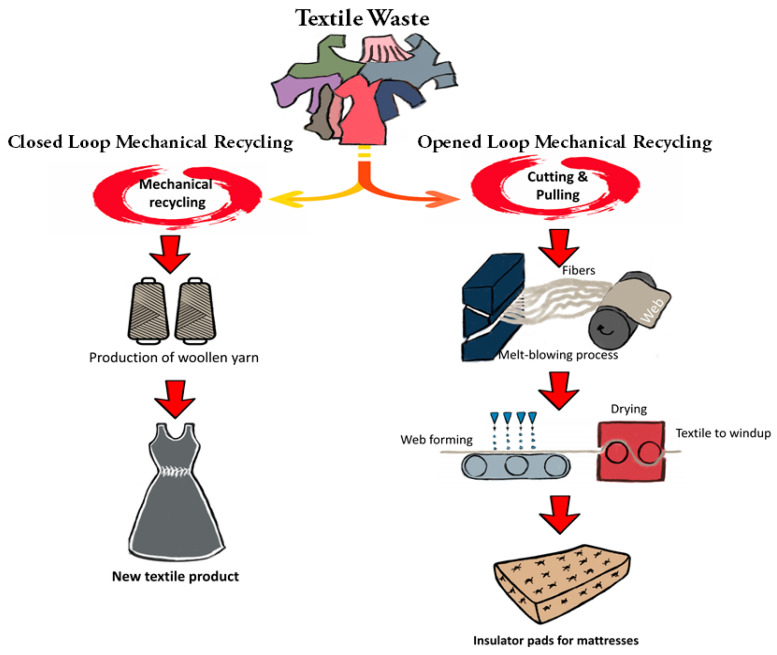
The recycling of garments by open and closed-loop systems.

Figure 4 shows an open and closed-loop garment recycling. The term “open-loop recycling” refers to methods in which fabrics or textiles are used to make new products [52]. Besides that, recycling wool through open-loop recycling can be used to mechanically extract the garments back into a fibrous form, and these materials can be used as raw materials. Non-woven fabrics are frequently made from these materials by garneting, carding, or air laying webs, which are then mechanically, thermally, or chemically bonded. Furthermore, hot water converges with the fibers when the melt-blown process is applied, followed by spun lace. The purpose of spun lace is to entangle fibers. The method utilizes extremely tiny high-velocity water jets rather than sharp needles when the textile is going through the drying process [97]. Besides that, the melt blowing process can be combined with polymer melt extrusion passed by the attenuation of the extrudate and orifices under extremely high-velocity air jets to generate nonwoven fibers in just one step [98].

In addition, Tsai described a technology to develop an electret via saturating a nonwoven textile with a liquid and then removing the fluid. This technology contributed (i) a sheet made from a thermoplastic fiber nonwoven web; (ii) the ability to place the sheet underneath a roller: water jets were added in these steps to acquire a saturated sheet, and (iii) a suction process that is able to remove the water at the saturated sheet that then allows it to dry [99]. The applications of open-loop recycling include insulators for mattresses and sound absorption [97]. Nylon waste, such as fishing nets (nylon 6) and carpets, is the common raw material for that is used in mechanical recycling. Nylon materials are recycled via reshaping and melting to create new fibers with appropriate strength and length [100].

Closed-loop textile recycling refers to processes in which the recycled material is identical to the provided material. Both pre-and post-consumer mechanically recycled materials can be considered to be closed-loop recycled since the waste material or fiber re-enters the apparel generation chain. The fineness, length, polymer quality, and shading of the new fiber properties can determine the quality and to create the most appropriate final product [48]. A closed loop conducts the recycling of wool fibers. Using traditional techniques, recycled wool yarns can be turned into very high-value apparel products. Woolen materials are mainly formed from long fibers that have been carefully treated, allowing for mechanical recycling to covert the wool into fibers. Wool recycling follows the same methods as mechanical cotton recycling [101]. The Cardato brand coat from M&S Shops is manufactured using closed-loop recycled wool. The recycled wool waste should ideally be compatible with the same carding, spinning, and fabric production processes used to process raw wool during the production of woolen yarn [101,102,103]. The post-consumer wool recycling can be applied. This depends on effective strategies to reduce fiber breakage and to maximize residual fibers length following the mechanical pulling method being available [104].

Klaus-Nietrost et al. studied the recycling of cellulose-based textiles with a minimum of one synthetic plastic. The regeneration process was applied to acquire a cellulosic-molded body. In addition, the cellulosic-molded body was composed of a crucial substance in the form of lyocell—fibers or viscose—fibers. Chemical fibers, viscose fibers, and regenerated fibers are all terms that are used to describe different types of fibers. They can be produced using the viscose technique, which is a wet-spinning method [105,106]. The cellulosic material is soaked in N-methyl morpholine-N-oxide (NMMO) and is pressed with a spinneret. It coagulates and generates a new fiber [107]. When recycled fibers are applied in cellulose production, the main issue is the purity of the raw materials, such as old-fashioned textile materials, and more significantly, the contamination of synthetic polymer materials. Nevertheless, the synthetic polymer can be denoted as being combined with cellulose materials [105].

## 6. Chemical Recycling of Textile

Chemical treatment is seen as a promising textile recycling method. During this process, chemicals are added to degrade the complex textile polymer to create smaller polymer molecules. The “CRM”, or chemical recycling to monomer, is a popular nowadays that can be used to degrade polymers such as those in textile waste to monomers. Furthermore, a solution-purified polymer, an oligomer, a monomer, or a crude feedstock in gaseous or liquid form are all examples of textile chemical recycling products. Re-polymerization can be used to renew polymers such as oligomers and monomers [90,108]. Table 2 lists the industrial scale that is applied when recycling textiles through various chemical processes. Typical chemical processes are glycolysis, hydrolysis, and alcoholysis. In addition, thermo-chemical methods consist of hydrocracking, pyrolysis, and gasification, which are typically conducted in the presence of catalysts at high temperatures [109]. Meanwhile, biochemical processes use enzymes or microorganisms in recycling processes such as enzymatic hydrolysis [106].

The drawbacks and advantages of the various chemical textile recycling processes are shown in Table 3. Chemical polymer recycling for cellulose-based synthetics and mixtures of textile fibers has been studied. Textile fibers are destroyed by mechanical methods, such as shredding, and their destruction is followed by a chemical dissolution process. Hazardous solvents are often used; the polymer is retained while the fibers are regenerated and spun [119]. In addition, the dyes need to be removed from the textile waste, and they are removed using harmful chemicals such as bleach. Ionic liquid solvents have recently been used to reintroduce dyed post-consumer textile waste into a new life cycle and to reduce environmental effects [120]. Furthermore, the conversion of cotton to viscose is one of the most well-known examples of chemical textile recycling. Pure cotton fabric is depolymerized into a pulp, which is transformed to viscose. The production process is similar to that made from wood pulp. Industry leaders such as Lenzing and Birla Cellulose adapted the conversion of cotton to viscose technology, with the beginning of the process being the depolymerization of 100% cotton textile materials. Nevertheless, the yield of the synthesized polymer chains is lower than that of the pure wood pulp process due to decreased physical properties. Therefore the regenerated fibers must be combined with virgin viscose fibers to achieve a high yield [121].

Nylon 6 is a polyamide made from the seven-membered ring caprolactam, making it a valuable candidate for CRM. Since the early 1960s, researchers have been studying the depolymerization of nylon 6 back to a monomer, and CRM has been used for nylon 6 for decades [122]. The reactor is conducted by superheated steam and a catalyst to form a caprolactam distillate. Nylon 6 is made from crude caprolactam that has been distilled and depolymerized. In terms of purity, the caprolactam obtained is similar to virgin caprolactam. The repolymerized nylon 6 is spun into yarn and is tufted into carpet. The physical properties of the carpets produced using this technology are fairly similar to those of virgin caprolactam carpets [123,124,125]. In addition, textile waste can be used to produce geotextiles (thick ropes), which can be used to protect slopes from erosion and sliding. Natural fibers are dissolved with mixtures of 5% sodium hypochlorite (NaClO) and Schweizer’s reagent for cellulosic fibers as solvents. Then, the geotextiles are installed in conditions where the ground is sloped and where there is vegetation—this method reduces soil movement and pedestalling. [126,127]. Another application for textile waste that has been processed by chemical recycling is for use as packaging. The cellulose nanofiber textile waste has been used to enhance the strength performance of biopolymers. The combination of polylactic acid and cellulose nano-fibrillated fiber (CNF) for packaging applications has shown excellent strength reinforcement ability, tensile modulus, and elongation [128,129].

**Figure 5:**
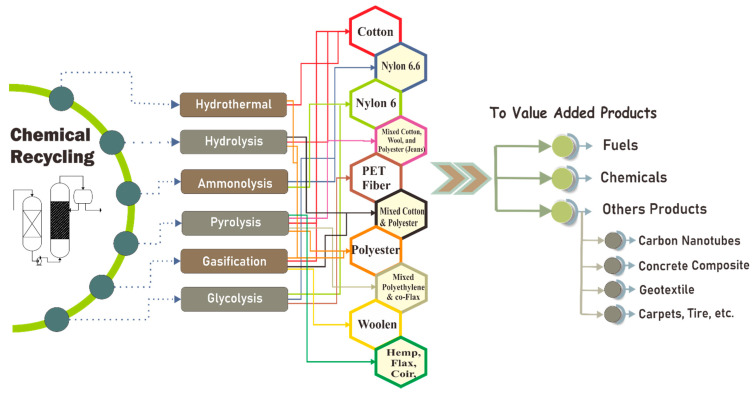
The possible pathways to recycling textiles using chemical methods.

The chemical recycling of textiles usually necessitates integrated material flow and energy within the chemical industry. Nevertheless, some sorted waste products from municipal solid waste have more potential for small-scale feedstock production [130]. Figure 5 shows the possible pathways to recycling textile using chemical methods. Furthermore, Yousef et al. studied a long-term approach for recovering cotton from textile waste and regenerating it to become a new substance. The primary processes used to recover the cotton were leaching nitric acid, dissolving dimethyl sulfoxide, and diluting hydrochloric acid for bleaching. This recovery rate, carbon footprint, and economic performance of this technology were determined to be 93%, −1534 CO_2_ eq/ton, and 1466 USD/ton, respectively [73] if textile waste is used as a renewable resource for the production of thermal energies. Nunes et al. showed that cotton briquettes had a heating value of 16.80 MJ/kg and a cost of 0.006 EUR/kWh when used as a fuel [131].

### 6.1. Textile Recycling Using Pyrolysis

Textile recycling using pyrolysis is a promising technology that can be used to degrade the carbon-polymers of solid waste in order for them to become three pyrolysis products in the solid, liquid, and gas states [140,141,142]. Pyrolysis could be utilized in diverse textile materials that have not been sorted prior or in multi-material textile products that otherwise would have only been treated in waste-to-energy facilities [143]. Under oxygen-free circumstances, pyrolysis reallocates C/H/O elements from organic molecule waste into three-phase pyrolysates. Syngas (a combination of H_2_ and CO) produced by thermochemical processes was utilized as a direct fuel or as a raw material to produce other hydrocarbons and alcohols [144,145,146].

The pyrolysis process does not require pretreatment, and it is a potential method for treating polluted waste. These factors make pyrolysis a more efficient process than chemical methods (including bio-chemicals), which require many chemicals and frequently produce a lot of trash that must be re-landfilled and takes longer to operate on a large scale [147,148,149]. Table 4 shows a summary of the diversity yields of the solid, liquid, and gas products produced via textile waste pyrolysis. Commonly, the liquid products produced from textile waste are mono-polyaromatic and oxygen compounds containing hydrocarbons, such as alcohols, aldehydes, ketones, and carboxylic acids [150]. The production of oil and tar formation from the pyrolysis process depends on raw materials and operation conditions [151].

The aromatic oxygen and hydrocarbon compounds were found at 500 °C. Furthermore, when the temperature reaction was as high as 600 °C, alkylphenols were produced, and at a temperature greater than 800 °C, the yield of the oxygen compounds began to decrease slowly. The condensable compounds at 800 °C were predominantly aromatics with no substituent groups, benzene, or naphthalene. On the other hand, if the temperature reaction was about 850 °C, the aromatic hydrocarbon compounds of 3 and 4 rings were produced [152].

Char derived via textile waste pyrolysis can be applied as a primary filler or a hybrid filler (carbon nanotubes/nanoball (CNTs/CB)) or as graphene oxide (GO/CB) in concrete composite applications. To synthesize CB of 10–20 nm, the process is conducted in a pyrolysis reactor followed by milling and chemical treatments to modify the cotton textile waste (CTW). Then, synthetic CB (0.05 wt%) and hybrid fillers (CNTs/CB and graphene/CB: 50/50) are applied to improve the cement paste characteristics [153]. On the other hand, Jagdale et al. studied applications of recycled and reused carbon produced from cotton waste by the pyrolysis process in active electrode battery materials. The cotton-based carbon fiber electrode demonstrated outstanding cycling behavior and provided a high discharge capacity, with a voltage range of 0.02–1.2 V [154].

Furthermore, pyrolytic char derived from cotton waste can also be utilized as an adsorbent. A one-step low-temperature pyrolysis process produced char-based adsorbents made from CTW with different iron salts compounds. The Freundlich model fit the adsorption processes well when measuring Cr(VI) the adsorption performance of the chars, and char-FeCl_3_ had the highest adsorptive capacity of 70.39 mg/g. Consequently, this low-temperature pyrolysis method was cost-effective and straightforward to implement, with a high adsorption capacity for Cr(VI) removal [158]. The catalytic pyrolysis of nylon 6 waste fishing nets (WFNs) over a ZSM-5 zeolite catalyst was investigated by Eimontas et al. The activation energy using ZSM-5/WFNs was larger than 112 kJ/mol via the free kinetic analysis method. The major volatile organic compounds were alkyl C–H, carbonyl (C=O), and caprolactam [159].

Silva et al. studied colored cotton wastes to generate renewable aromatic hydrocarbons by the catalytic reforming of ash pyrolysis utilizing a pyrolysis raw material (colored cotton wastes). These materials have high energetic compactness, which is good for use thermochemical processes. Light oxygenated carbon chains with up to four carbons resulted from the ash pyrolysis. The partial deoxygenation and cracking reactions were increased by pyrolysis vapor reformation at 500 °C, which could be improved by catalytic reforming [160].

The catalytic pyrolysis over Ultrastable Y (USY) zeolite was investigated by Wang et al. This method produces benzene, toluene, and xylene (BTX) via Diels–Alder reactions by means of catalytic co-pyrolysis with specified plastic wastes and furanic substituents via the catalytic deconstruction of textile waste. The selectivity of co-pyrolysis to BTX for polyethylene and 20% co-flax waste was 81.6% and 80%, respectively [161]. In addition, the primary components of dryer lint were produced when the drying garments were cotton and polyester. The range of the activation energy (Ea) of lint pyrolysis was 167–204 kJ/mol, and the primary products for a heating rate of 25 °C/min were furan, 3-methyl- (4.78%), furfural (6.48%), isobutane (10.77%), and carbon dioxide (11.68%) [162].

### 6.2. Textile Recycling Using Enzymatic Hydrolysis

Hydrolysis is a chemical breakdown process that occurs due to a reaction with water. Generally, the hydrolysis process requires a pretreatment process using acids, alkaline, and ionic liquid. Pretreatment is crucial in hydrolysis because it can affect the yield percentage [163]. Damayanti and Wu have already described recycled PET hydrolysis [109]. Before the recycling pf PET fibers from textile waste takes place, the pretreatment process is required. Pretreatment methods reduce the complexity of the structures of the textile materials by eliminating unwanted contaminants and improving hydrophilicity. PET fibers can be converted into raw materials through the following processes or reactions [164]:

The pretreatment process for textile waste materials that uses bases such as sodium, potassium, calcium, and ammonium hydroxides is known as alkali pretreatment. Alkali pretreatment will improve lignin solubilization and decreases cellulose crystallinity by enhancing cellulose digestibility. As a result, alkali pretreatment produces a high glucose yield. The reduced formation of fermentation inhibitors is a primary benefit of alkali pretreatment [165,166]. Furthermore, pretreatment using ionic liquids is a promising method due to the use of molecules that are more environmentally friendly that can dissolve cellulose at moderate temperatures without degrading the cellulose or the solvent. Solvent recovery can be applied through a thermal treatment with lower pressure [167]. The ionic liquid from organic solvents such as N-methylmorpholine-N-oxide (NMMO) and 1-allyl- 3-methyl-imidazolium chloride ([AMIM]Cl) was effective in pretreating cotton waste under enzymatic hydrolysis [168].

On the other hand, acid pretreatments such as sulfuric and phosphoric acid have been examined by researchers, even in the presence of cellulose solvents [169,170]. By breaking the polymeric structures in hemicellulose, acid pretreatments can hydrolyze the polymeric structures into monomers by increasing the availability of cellulose, hence boosting biodegradability [171]. There are some challenges such as the formation of side products (furfural), the cost of acid being relatively expensive, and the requirement of corrosion-resistant equipment [172].

Cotton waste from the textile industry has been discovered to be a viable feedstock for cellulosic ethanol production. Cotton waste can pre-treated and acid hydrolyzed to convert cellulose to reducing sugars. These findings are promising for ethanol production [173]. Table 5 lists the diverse range of textiles that can be used to generate glucose via hydrolysis. Furthermore, Pd-Au/SiO_2_ bimetallic catalysts are effective in oxidizing glucose generated from cotton waste. These experiments were conducted under the correct temperature and pressure operating conditions for acidic hydrolysis [174]. The recovery of polyester and sugar compounds from textile waste via enzymatic hydrolysis was investigated by Li et al. The temperature of the pretreatment process was −20 °C for 6 h, with the concentration of NaOH/urea of 7%/12%. The glucose recovery was 98.3%, with a cellulase dosage of 20 FPU/g [47].

Cellulases act as catalysts in hydrolysis reactions. In theory, these enzymes are unaffected by the reaction equilibrium and can be used again and again. Unfortunately, because these enzymes are massive organic compounds, some decomposition processes that occur over time must be considered [179]. Furthermore, these enzymes can break down material structures to change their physicochemical properties and biodistribution [180,181,182]. Enzymatic reactions involving various selections of materials can be initiated by certain enzymes [180,183]. As a result, the enzymatic breakdown of certain portions of these materials may be selective [184]. A commercial cellulase mixture typically contains three types of enzymes, each of which performs a different role in the catalytic process: (i) *Endoglucanases* degrade the cellulose chain at varying stages along the chain by increasing the number of accessible end parts, (ii) *Exoglucanases* degrade *cellobiose* units from both ends of the chain, and (iii) the depolymerization of the *disaccharide cellobiose* into monosaccharide units, e.g., glucose is carried out by *beta-glucosidases*. These three types of enzymes have generated excellent degradation performance in previously reported experiements [185].

Fermentation is an enzymatic degradation procedure for recovering textile waste such as cotton, polyester, nylon, and silk. For bioethanol production, five fermentation techniques can be implemented to degrade textile polymers: (i) Simultaneous saccharification and fermentation (SSF) converts sugars to ethanol [186]. Many researchers have used engineered microbes and have cultured them in biomass sugar solutions, such as glucose, xylose, mannose, galactose, and arabinose. The most commonly utilized microbes for ethanol production include *Saccharomyces cerevisiae, Zymomonas mobilis*, and *Aspergillus niger* [187]; (ii) separated hydrolysis fermentation (SHF) is a process where hydrolysis and fermentation are separated to produce bioethanol. SHF is a typical approach in which hydrolysis takes place first, followed by the fermentation process. This method allows the hydrolysis process to generate monosaccharide sugar initially, ensuring that sugar is available when fermentation starts [186,188,189]; (iii) semi-simultaneous saccharification and fermentation (SSnF): the excess sugar that accumulates during hydrolysis reduces enzyme activity, slowing down the process [186,190,191]. The short pre-hydrolytic method is used before the SSF process in SSnF. In addition, the yield of bioethanol production is be slightly higher than it would be if traditional SSF procedures were used [191]; (iv) consolidated bioprocessing (CBP) is required to degrade refractory biomass materials into solubilized sugars. Because CBP combines three processes (enzyme synthesis, saccharification, and fermentation), it is a viable technique for successful biofuel production. This technique can lower the cost of the reactor and enzymes, two main roadblocks to low-cost biomass processing [192,193]; (v) *submerged fermentation (SMF)* is one of the fermentation techniques that focuses on enhancing cellulase production. It is a widely accepted fermentation process for the synthesis of industrial enzymes because it is easy to regulate all of the factors such as pH, temperature, and operational approaches [186,187].

Table 6 lists ethanol production from various textile wastes using enzymatic hydrolysis and microbe fermentation. Jeihanipour and Taherzadeh studied ethanol production from the cotton from waste fabric. The alkali pretreatment was conducted in the concentration of NaOH (12 wt%) at 0 °C for 3 h, which was then fermented to ethanol via *Saccharomyces cerevisiae,* with an ethanol yield up to 99.1% [194]. Sanchis-Sebastiá et al. investigated green liquor as an alkaline solution in the processing of textiles to connect textile recycling with pulp mills. The textile recycling efficiency was predicted be 70% when using *Cellic CTec 2* as a hydrolysis enzyme. Nevertheless, the sodium reduction in the pulping process would be less than 15% [195]. On the other hand, Sebastia et al. studied the cotton fiber recycling process when it was conducted at 130 °C via acid hydrolysis. Sulfuric acid could depolymerize the structure of cotton to obtain glucose. The yield was over 90% when a two-step method was used [196].

### 6.3. Textile Recycling Using the Hydrothermal Method

The hydrothermal method is a decomposition process using chemical crystallization engineering techniques at high temperatures and pressures, with water as the main constituent of the reaction. The hydrothermal method is one of the most promising alternative methods for degrading carbon-polymer waste and organic components into three product phases: liquid, solid, and gas phases. It is conducted in an autoclave reactor in which organic acids can catalyze [200]. The hydrothermal process does not require pretreatment and is a method that utilizes water and can be classified into five groups based on the temperature range that is used: (i) hot water extraction, (ii) pressurized hot water extraction, (iii) hot liquid water treatment, (iv) hydrothermal carbonization, and (v) hydrothermal liquefaction. The method requires temperatures that are less than 280 °C [201]. Table 7 lists a summary of hydrothermal products produced via textile recycling. The hydrothermal conditions control the powder formation and particle size. The drawbacks of the hydrothermal process are the high temperatures, high pressure, and long reaction time that are required for the material manufacturing process [202].

Hongthong et al. studied the conversion of nylon 6 fishing net waste in the hydrothermal liquefaction of the macroalgae *Fucus serratus*. The hydrothermal liquefaction of macroalgae was performed for 10 min with a temperature reaction of up to 350 °C. The scope of nylon polymers included nylon 6 and nylon 6,6. A bio-crude yield of up to 17 wt% could be with a combination of 50% nylon 6 and *F serratus*. Furthermore, nylon 6 fully degraded in the process that produced the molecule caprolactam [203]. On the other hand, Xu et al. determined that CTW was catalyzed using surfactants during hydrothermal carbonization. The application of Span 80 and sodium dodecyl benzenesulfonate enhanced the conversion of CTW into bio-oil. Nevertheless, Span 80 was better for the formation of pseudo-lignin, which gave the produced hydro-chars a higher energy density and improved their fuel quality and combustion performance. The majority of bio-oil yields are aromatic chemicals, acids, hydrocarbons, ketones, and esters, with yields of up to 12.79%, 11.3%, 25.4%, 23.09%, and 14.88%, respectively. In addition, cyclopentenone substances (3-methyl-2-hydroxy-2-cyclopentene-1-one and 3-ethyl-2-hydroxy-2-cyclopentene-1-one) were also found [135]. H_2_SO_4_ successfully degraded CTW through hydrolysis [204].

When recycling cotton-polyester textile waste with an organic acid catalyst, the hydrothermal process includes two steps: (i) splitting the mixture of polyester–cotton fiber waste into pieces and (ii) dispersing the mixture an organic acid catalyst in an aqueous phase to produce products. The reaction temperature was up to 140 °C under high pressure. The recycling yield of the obtained polyester fiber aggregate was 99%, and the recycling yield of the cotton fiber fragments was 81% [213]. The cotton was dissolved easily in a citric acid solution, and it was degraded in a strong acid solution. On the other hand, the cotton was unaffected by strong alkaline solutions [211]. Qi et al. used FeCl_3_ as a catalyst to increase cotton textile waste conversion to hydrochars, achieving highly efficient hydrophobicity by lowering the hydrothermal carbonization temperature. In addition, the simultaneous impacts of FeCl_3_ and hydrothermal carbonization produced a side reaction pathway by allowing more furfural compounds as derivatives [134].

### 6.4. Textile Recycling Using Ammonolysis

The ammonolysis process is the primary depolymerization method of nylon 6,6 carpet waste. Mckinney studied the reactions of nylon 6,6 and nylon 6,6/nylon 6 mixtures with ammonia or ammonium phosphate as a catalyst. The temperature and pressure of this process were 300–350 °C and 68 atm, respectively. The major products resulting from the depolymerization of nylon 6,6 were 5-cyanovaleramide, hexamethylenediamine, and adiponitrile. Furthermore, 6-aminocapronitrile, ε-caprolactam, and 6-aminocaproamide were the primary products of nylon 6 [214]. The depolymerization mechanism of the nylon 6,6 and nylon 6 mixture was described by Kalfas et al. The amine chain would have broken down, which would then be followed by the dehydration process and the ring addition and ring-opening reactions for the presence of cyclic lactams in nylon 6 [139].

### 6.5. Textile Recycling Using Gasification

Textile recycling by means of gasification processes is conducted under temperatures up to 400–1000 °C. The reactions occur under low-oxygen or oxygen-free conditions [152,215]. Compared to pyrolysis, gasification is more complicated since chemical reactions occur on the substance [216]. The gasification approach generates gases with high carbon and hydrogen concentrations, similar to the pyrolysis process. Nevertheless, the gaseous fraction in gasification is dramatically higher than that in pyrolysis [216]. The increasing pressure can lead to higher yield, calorific value, CO, and H_2_ concentrations when using gasification [217]. In contrast, the syngas output increases when the operating temperatures rise, whereas the char yield lowers [218]. Gasifiers can be clarified based on the reactor bed and flow type. Fixed bed and fluidized bed gasifiers are the most common gasification process. On the contrary, the spouted bed gasifier is a unique case of a fluidization type. Low gas flow rates can manage coarser solid particles, although segregation is hampered by a unique hydraulic structure [219].

Synthesis gas, often known as syngas, is the desired end product in gasification, and includes compounds such as hydrogen, carbon dioxide, carbon monoxide, and methane (CH_4_). The by-products of ethylene and ash can be formed [151]. Vela et al. studied the steam gasification of mixed textile waste using a fluidized bed bench-scale reactor at the reaction temperature up to 850 °C. The yields of CH_4_ with pure polyester, cotton, and a mixed (50% polyester and 50% cotton) were 7.0%, 3.4%, and 10.7%, respectively [220]. Furthermore, the textile wastes that were used were cotton, wool, and polyester fibers with activation energies of 144.49, 79.37, and 178.15 kJ mol^−1^, respectively. Char gasification took place in the temperature range of 800–1000 °C, with the primary product being CO_2_ [221]. On the other hand, textile waste activation energies (cotton, woolen, polyester fiber) up to 89.0 kJ/mol were achieved with a 5 wt% Fe_2_O_3_ catalyst [222].

### 6.6. Textile Recycling Using Glycolysis

Glycolysis is a degradation process that can be used to convert the large molecules of a textile to small molecules. This process is widely used to recycle textile materials such as PET fiber and polyurethane. PET fiber is the most widely used textile fabric in the textile industry due to its outstanding mechanical characteristics and inexpensiveness. Consequently, the generation of PET waste is also very large [223,224]. The large volume of PET fiber waste has wreaked havoc on the ecosystem. It is essential to find a sustainable and economical technique for recycling PET fiber waste to protect our resources and the environment. The glycolysis process has many advantages: it has a short reaction time and uses minimal energy. Nevertheless, PET fiber of glycolysis is slowly processed without a catalyst [225,226].

For degradation without a catalyst, it is challenging to glycolyze PET waste. A lot of effort focuses on developing a high-performance and eco-friendly catalyst to degrade the macromolecules of textile fibers to bis(2-hydroxyethyl) terephthalate (BHET) monomers and the other chemicals [227]. The recycling of polyester textiles with Mg-Al double oxides as a catalyst was studied by Guo et al. The yield of BHET was over 82 mol% using a Mg-Al double oxide catalyst with wet mixing or co-precipitation. Furthermore, the recycled PET (r-PET) fibers that were formed from the re-polymerization process had similar spinnability and mechanical characteristics to virgin PET fibers [228].

The depolymerization process of nylon 6 via the glycolysis process was investigated by Hommez et al. [229]. The glycolysis process was conducted at 250 °C in the presence of phosphoric acid. The free carboxylic acid and carboxylic acid were esterified with ethylene glycol; the major products were caprolactam, N-(5-hydroxy-3-oxa-pentyl)-caprolactam, N,N’-ethylene-di(caprolactam), and N-(2-hydroxyethyl)-caprolactam, and linear oligomers were reported. Huczkowski et al. investigated the glycolysis of nylon 6 in boiling ethylene glycol with and without a catalyst, resulting in oligoamides containing amino- and hydroxyl end-groups [230,231]. Holland and Hay presented the thermal degradation of nylon 6 and nylon 6,6 to produce a crosslinking formation and non-volatile char [232]. Kim et al. studied the glycolysis of nylon 6,6 with ethylene glycol to obtain major products from the bis(β-hydroxyethyl)adipate and β-hydroxyethylester group [233].

The glycolysis of polyester fibers is traditionally conducted in a boiling ethylene glycol solution under atmospheric pressure with several metal catalysts, e.g., zinc acetate (Zn(Ac)_2_). Zinc acetate can be used as a catalyst in the glycolysis process. The operation conditions for this process are 1 h and 96 °C. The PET fiber conversion and monomer yield of BHET achieved 100% and 80%, respectively. The oligomers were broken down quickly into dimers, which caused an instability process, resulting in a low molecular weight [234]. On the other hand, the glycolysis process of PET fibers to produce BHET with acetic acid, lithium hydroxide, sodium sulfate, and potassium sulfate resulted in yields of up to 64.42, 63.50, 65.72, and 64.42%, respectively [235].

### 6.7. Decolorization Technology in Textile Recycling

Decolorization technology is a critical problem in the high-quality chemical recycling and recovery of textile wastes. Only 1% of textile wastes, mainly white-colored wastes, are recycled, so the final color quality of regenerated fibers is uncontrollable. Color removal is required for the large-scale circulation of non-thermoplastic fibers Technologies for color removal from textile wastes are dye destruction or extraction, which take place during the pre-recycling process. Dye destruction methods, such as oxidation and photodegradation, may damage polymers and change the dyeability of the regenerated textiles [236]. To lower the chemical potential of the dyes in solution, harsh extraction conditions such as ultra-high temperature (>150 °C), lengthy time, and the use of excessive corrosive solutions can lead to structural damages to the polymers. Dye extraction focuses on finding solvents with high dye solubility [237], but dye extraction fails to remove all of the dye from textiles. Mu and Yang demonstrated the minimization of fiber density by solvents and temperatures, completely removing dispersed dyes, acid dyes, and direct dyes from PET, nylon, and cotton fibers, respectively [238].

When color removal takes place after textile degradation, the final colors are not predictable due to the uncontrollable mixture of colors in the collected textiles, for example, when cotton with multiple shades is dissolved in ionic liquids to spin regenerated cellulose fibers [239]. Li et al. used nitric acid-modified activated carbon (AC-HNO_3_) as an adsorbent for the removal of C.I. Disperse Red 60 (DR60) from the glycolysis products of waste PET fabrics and for the rapid decolorization of textile dyes using ceria and tin-doped ZnO nanoparticles [240,241]. Huang et al. presented ion-exchange resin (D201) to efficiently remove colorants after PET glycolysis [226]. The removal rate of the colorant and the retention efficiency of BHET was over 99% and 95%, respectively. Huang et al. presented a modified recrystallization process using ethyl acetate as the solvent needed to remove colored impurities from BHET. The process showed a decoloring rate of over 97.5% for the model-colored impurities, performing better than the reported physical adsorption processes [242].

## 7. Conclusions

The fashion industry faces some challenges in implementing a CE; it must consider environmental laws, policies, and the CE’s long-term viability. Digital innovation and industry 4.0 have significantly impacted CE transformation with data-driven product lifecycle analysis. By implementing IoT and RFID technology, physical production processes, such as material transportation, and the associated information flows, such as the visibility and traceability of numerous manufacturing operations, can be easily connected. Furthermore, the recognition rate of textile waste can be achieved with up to 100% accuracy using NIR technology. Mechanical and chemical recycling are common methods that take place during the textile recycling process. Textile materials such as cotton and wool can be converted into useable yarn (re-woven or knitted) by spinning carded yarn and mixed shoddy. In addition, garnering, carding, or air laying webs, which are subsequently mechanically, thermally, or chemically bonded, can be widely used to make non-woven fabrics from these materials. On the other hand, chemical treatment is seen as a promising method for textile recycling. Chemical textile recycling can be applied to degrade textile waste into feedstock monomers. Cotton waste from the textile industry has been identified as a suitable feedstock to produce cellulosic ethanol with a yield of more than 90% by means of enzymatic hydrolysis. It may be worthwhile for future experimental studies to investigate chemical recycling methods that use enzymatic hydrolysis with an ionic liquid to obtain high-value fibers. Nevertheless, the toxicity of the dissolving solvents is still a major concern; the amount of energy and water that are consumed are affected by environmental issues. Furthermore, real-time sensors to identify various types of textile materials are needed.

## Figures and Tables

**Table 1:** The recognition rate with different types of analyzers.

Textile	Analyzer	Mathematic Algorithms	Recognition Rate, %	Ref.
Pure (polyester slash; wool; cotton; polyester normal; nylon). Mix (polyester/nylon; polyester/wool; polyester/cotton slash; polyester/cotton).	NIR	SVM, MLP + CNN	92–98	[74]
Cotton, polyester, polyamide, acrylic, silk, wool.	NIR	SIMCA	93–97	[79]
Polypropylene, polyethylene terephthalate, polylactic acid, cashmere, Tencel, cotton, wool	NIR	PCA + SIMCA	100	[80]
Wool	NIR	PLS, ECR		[81]
Satin, twill, plain	ResNet-50	CNN	99.3	[82]
Satin, twill, plain	Epson Scan V330	TILT, HOG, FCM clustering, histogram equalization data	94.5	[83]
Non-woven fabric, plain weave, twill weave, double jersey, satin weave	Canon scanner 9950F, Self-organizing map (SOM) network	CIE + Co-occurrence matrix	92.6	[84]
Cotton, polyester, viscose	NIR	NA	76–100	[85]

CIE: C-Illuminant (light source C), FCM: fuzzy C-means, HOG: histogram of oriented gradients, PCA: principal component analysis, SIMCA: soft independent modeling of class analogy, SOM: self-organizing map, SVM: support vector machine, TILT: transform invariant low-rank textures. PLS: partial least squares. ECR: elastic component regression. NA: not available.

**Table 2:** Various chemical processes applied in textile recycling at the industrial scale.

Company	Material	Method	Country	Ref.
Ambercycle	Textile	Enzymatic treatment	Los Angeles	[110]
BlockTexx	Cotton and polyester (PET)	Fiber separation technology	Australia	[111]
FENC	PET and textile	Hydrolysis	Taiwan	[112]
Infinited Fiber	Cellulose and polyester (PET)	Alkaline extraction	Finland	[113,114]
Ioncell	Cotton and polyester (PET)	Ionic liquid solvent polymer dissolution	Finland	[115]
Lenzing	Pre-consumer cotton and post-consumer garments	Closed loops and chemical recovery	Austria	[116]
Tyton BioSciences	Cotton pulp, polyester (PET), poly-cotton blends	Subcritical water treatment	Danville	[117]
Worn Again Technologies	Cotton and polyester (PET)	Dissolution polymer Solvent separation	UK	[118]

**Table 3:** The drawbacks, advantages, and disadvantages of chemically recycling textiles.

Type of Technology	Advantages	Disadvantages	Ref.
Pyrolysis	▪ Simple process ▪ Many different kinds of raw materials can be used for the pyrolysis process.	▪ High-temperature reaction process ▪ High energy consumption	[132,133]
Enzymatic Hydrolysis	▪ The novel microorganism enhances the production of biopolymers and conversion rate. It can effectively be spun into novel waste textile fibers ▪ Under mild conditions ▪ Low energy demand ▪ Utilizes environmentally friendly solvents and chemicals	▪ Only possible to recycle certain materials using enzymatic hydrolysis such as rayon, cotton, hemp, etc. ▪ Needs large amounts of water	[120]
Hydrothermal	▪ Low ash ▪ High heating values ▪ Relatively low temperatures than gasification and pyrolysis processes ▪ Reduced oxygen content	▪ Long reaction time ▪ Lower purity and heterogeneity	[134,135,136]
Gasification	▪ Potentially mixed textile waste	▪ High energy consumption ▪ High temperature is needed	[137]
Glycolysis	▪ Low energy consumption	▪ Low selectivity ▪ Without a catalyst, slow processing	[138]
Ammonolysis	▪ Various types of textile waste can be applied	▪ Generates a mixture of primary, secondary amines ▪ Applied toxic solvent (ammonia) ▪ High pressure and temperature	[139]

**Table 4:** Yields of solid, liquid, and gas products produced via textile waste pyrolysis.

Raw Materials	Reactors	Catalyst	Reaction Time, Min	Temperature, °C	Yield (%)			Major Products	Ref.
					Solid	Liquid	Gas		
Egyptian banknote ELCBs Cotton, 100%	Batch	NA	30	700	15.65	29.28	55.07	2-Propanone, toluene, 3-furaldehyde, 2-furalmethanol, benzaldehyde, phenol, acetophenone	[147]
Hemp, 100%	Batch	NA	120	750	24	48.5	27.50	Activated carbon	[155]
Flax, 100%	Fixed bed	ZnCl_2_	220	450	44.80	NA	NA	Activated carbon	[156]
Egyptian banknote ELCBs Cotton, 100%	Batch	NA	30	600	17.26	30.28	52.46	1,3-Dioxolane-2-propanal, 2-methyl, furfural, 2-furanmethanol, 4,4-ethylenedioxy-1-pentylamine	[147]
Flax, 100%	Fixed bed	ZnCl_2_	220	450	44.80	NA	NA	Activated carbon	[156]
Cotton, 70% Polyester, 30%	Batch	Dye-Originating Heavy Metals	35	500–700	17.79	37.59	44.62	Activated carbon	[147]
Egyptian banknote ELCBs Cotton, 100%	Batch	NA	30	500	20.74	39.75	39.51	Toluene, furfural, 2-furanmethanol, 2-furancarboxaldehyde, 5-methyl	[147]
Hemp, 100%	Fixed bed	ZnCl_2_	220	450	41.60	NA	NA	Activated carbon	[156]
Cotton, 100%	Fixed bed	Na_2_CO_3_	120	600	16.25	29.49	54.26	Furans, ketones	[130]
Coir, 100%	Fixed bed	NA	240	800	37.40	47.40	18.20	Activated carbon	[157]
Abaca, 100%	Fixed bed	NA	240	800	28.60	48.10	23.60	Activated carbon	[157]
Cotton, 100%	Batch	NA	70	700	12.50	74.00	13.50	Double bond carboxyl and carbonyl liquid	[143]
Low Grade biomass fiber (Flax, 100%)	Batch	NA	120	900	20	55	25	Activated carbon	[155]
Flax, 100%	Fixed bed	H_3_PO_4_	220	450	39.20	NA	NA	Activated carbon	[156]
Biomass fiber waste Jute, 100%	Fixed bed	NA	240	800	24.60	59.60	15.90	Activated carbon	[157]

**Table 5:** Conditions for converting textile fiber to glucose via enzymatic hydrolysis.

Textile Materials	Type of Pretreatment	Condition of Pretreatment		Enzyme	Condition of Hydrolysis		Glucose Yield, %	Ref.
		T (°C)	T (h)		T (°C)	T (h)		
Cotton, 90% Wool, 5% Polyester, 5%	Protease (Enzymatic)	50	48	*Cellic CTec3^®^ and Savinase 12T^®^*	50	70	95.0	[175]
Used Jeans	Phosphoric acid (Acid)	50	7	*Cellulase Trichoderma reesei and Aspergillus niger*	50	96	79.2	[176]
Cotton red T-shirt, 100% cotton black T-shirt, 100%	N-methyl-morpholine-N-oxide& Phosphoric Acid (Acid)	50	1	*Cellulase AP3, Aspergillus niger*	50	72	87.0–95.0	[177]
Cotton red T-shirt, 100% cotton black T-shirt, 100%	NaOH/Urea (Base)	50	1	*Cellulase AP3, Aspergillus niger*	50	72	48.0–55.0	[177]
Cotton T-shirts, 100%	1-allyl-3-methylimidazolium chloride (Ionic liquid)	110	1.5	*G. xylinus (Acetobacter aceti subsp. xylinus or A. xylinus)*	50	24	81.6	[168]
Towels, cellulose content 87.8%	Untreated Pretreatment	NA	NA	*Cellulase*	200	0.03	74.2	[169]
Waste blue jeans (polyester/cotton), 40%/60%	Sodium carbonate (Base)	150	2	*Celluclast 1.5 L and β-glucosidase*	45	72	81.71	[170]
Cotton, 100%	Sodium carbonate (Base)	150	2	*Celluclast 1.5 L and β-glucosidase*	45	72	88.0	[170]
Textile from End-of-life euro banknotes	NaOH/Urea (Base)	−20	6	*Cellulase*	50	382	96.0	[178]

**Table 6:** Ethanol production after enzymatic hydrolysis and microorganism fermentation of textile wastes.

Textile l	Type of Pretreatment	Hydrolysis		Enzyme for Hydrolysis	Fermentation Process		Microorganism	Type of Fermentation	EthanolYield, %	Ref.
		T (°C)	T (h)		T (°C)	T (h)				
Polyester/cotton (50%/50%)	N-methyl morpholine-N-oxide (Cellulose solvent)	NA	48	*Cellulase and β-glucosidase*	30	24	*Saccharomyces cerevisiae*	SHF	91.91	[197]
Polyester/cotton (40%/60%)	NaOH/Urea (Alkali)	45	72	*30 FPU C ellulase and 60 IU β* *-glucosidase*	36	72	*Saccharomyces cerevisiae*	SSF	70.00	[163]
Cotton, 100%	Na_2_S_2_O_4_ and Na_2_CO_3_ (Alkali)	50	48	*Cellulase AP3*	37	48	*Zymomonas mobilis*	SSF	90.00	[198]
Viscose/polyester (60%/40%)	N-methylmorpholine-N-oxide (Cellulose solvent)	NA	48	*Cellulase and β-glucosidase*	30	24	*Saccharomyces cerevisiae*	SHF	94.99	[197]
Cotton, 100%	Na_2_CO_3_ (Alkali)	45	72	*Cellulase and β-glucosidase*	32	24	*Saccharomyces cerevisiae CCUG 53310*	NA	69.40	[170]
Cotton ginning trash, 100%	Sulfuric acid (Acid)	50	96	*Cellic CTec 2 cellulase*	30	NA	*Saccharomyces cerevisiae*	SSF	70.00	[199]

SHF: separated hydrolysis dermentation, SSF: simultaneous saccharification and fermentation, NA: not available.

**Table 7:** Products and conditions of textile recycling using hydrothermal methods.

Feedstock	Reactor	Catalyst	Time, h	Temperature, °C	Major Product	Yield, %	Ref.
A blue cotton/polyester,	Batch	Hydrochloric acid	3	150	Cellulose powder	49.3	[205]
Waste cotton fabrics	Batch	Hydrochloric acid	1.6	150	Microcrystalline cellulose	85.5	[206]
Cotton/synthetic fibers,	Autoclave	NA	90	280	Volatile compound (CH_4_, C_2_H_4_)	98.0	[207]
Waste cottons	Hydrothermal reactor	β-cyclodextrin	3	250	Activated carbon	NA	[208]
Cotton textile waste	Tubular	Ferric chloride	1	700	Nanopowder	32.6	[209]
Waste cotton woven	Tubular	Ferric chloride	1.5	700	Activated carbon	NA	[210]
Light blue and white uniform with polyester/cotton,	Hydrothermal reactor	Citric acid	1	225	5′-hydroxymethylfurfural	12.5	[211]
Cotton	Semi-batch	Formic acid	1	250	Glucose	83.8	[212]

## Data Availability

No applicable.

## Abbreviations

AI	Artificial intelligence
BHET	Bis(2-hydroxyethyl) terephthalate
CBP	Consolidated bioprocessing
CE	Circular economy
CIE	C-Illuminant (light source C)
CNN	Convolutional neural network
CTW	Cotton textile waste
Ea	Activation energy
EG	Ethylene Glycol
FCM	Fuzzy C-means
HOG	Histogram of oriented gradients
ICT	Information and communication technology
IoT	Internet of Things
k-NN	k-nearest neighbors algorithm
LCA	Life cycle assessment
MLP	Multi-layer perceptron
NIR	Near-infrared spectroscopy
NMR	Nuclear magnetic resonance
PCA	Principal component analysis
PET	Polyethylene terephthalate
r-PET	Recycled polyethylene terephthalate
RMBs	Recycling business models
SAaaS	Software as a service
SHF	Separated hydrolysis fermentation
SIMCA	Soft independent modeling of class analogy
SMF	Submerged fermentation
SMOs	Smart manufacturing objects
SOM	Self-organizing map
SSF	Simultaneous saccharification and fermentation
SSnF	Semi-simultaneous saccharification and fermentation
SVM	Support vector machine
TILT	Transform invariant low-rank textures
WEEE	Waste electrical and electronic equipment
